# Supplementary material for: School-Based Intervention on Cardiorespiratory Fitness in Brazilian Students: A Nonrandomized Controlled Trial
Source: J Funct Morphol Kinesiol. 2019 Jan 21;4(1):10. doi: 10.3390/jfmk4010010 (PMC7739428; doi:10.3390/jfmk4010010)

Table S1. Sample size calculation.

Variables	Duration	IG	CG	ES	Sample		30%††	
		Δ	Δ		CG	IG	CG	IG
		Mean (sd)	Mean (sd)		*n*	*n*	*n*	*n*
BMI	-	-	-	0.37 * [39]	116	116	151	151
Flexibility (boys)	12 weeks	2.4 (8.9)	-1.2 (8.3)	0.45 ** [40]	79	79	103	103
Flexibility (girls)	12 weeks	3.2 (9.2)	0.7 (8.3)	0.35 ** [40]	128	128	166	166
Handgrip strength	6 months	2.7 (4.9)	4.4 (6.3)	0.30 ** [41]	174	174	295	295
Abdominal resistance	6 months	6.9 (1.2)	3.3 (10.0)	0.5 ** [41]	50	50	65	65
CRF	-	-	-	0.68 * [6]	35	35	46	46
Triglycerides	16 weeks	8.0 (9.7)	14.4 (12.5)	0.57 ** [42]	49	49	64	64
Total cholesterol	16 weeks	-19.2 (9.0)	1.9 (8.7)	2.38 ** [42]	4	4	5	5
HDL cholesterol	16 weeks	-8.5 (4.2)	-2.8 (5.1)	0.80 ** [42]	11	11	14	14
LDL cholesterol	16 weeks	-12.6 (7.0)	1.7 (7.5)	1.86 ** [42]	6	6	8	8
Body image (boys)	3ss × 60′	-	-	0.30 † [43]	139	139	181	181
Body image (girls)	4ss × 50′	-	-	0.48 † [44,45]	55	55	72	72

BMI: Body mass index; ES: Effect size; Sig: Significance; CG: Control group; IG: Intervention group; sd: Standard deviation; ss: Sessions; Δ: Difference between pre- and post-intervention; CRF: Cardiorespiratory fitness; HDL: High-density lipoprotein; LDL: Low-density lipoprotein; *Effect size estimated by meta-analysis; **Effect size calculated in G*Power 3.0 program; †Effect size showed in systematic review; ††Losses and refusals.

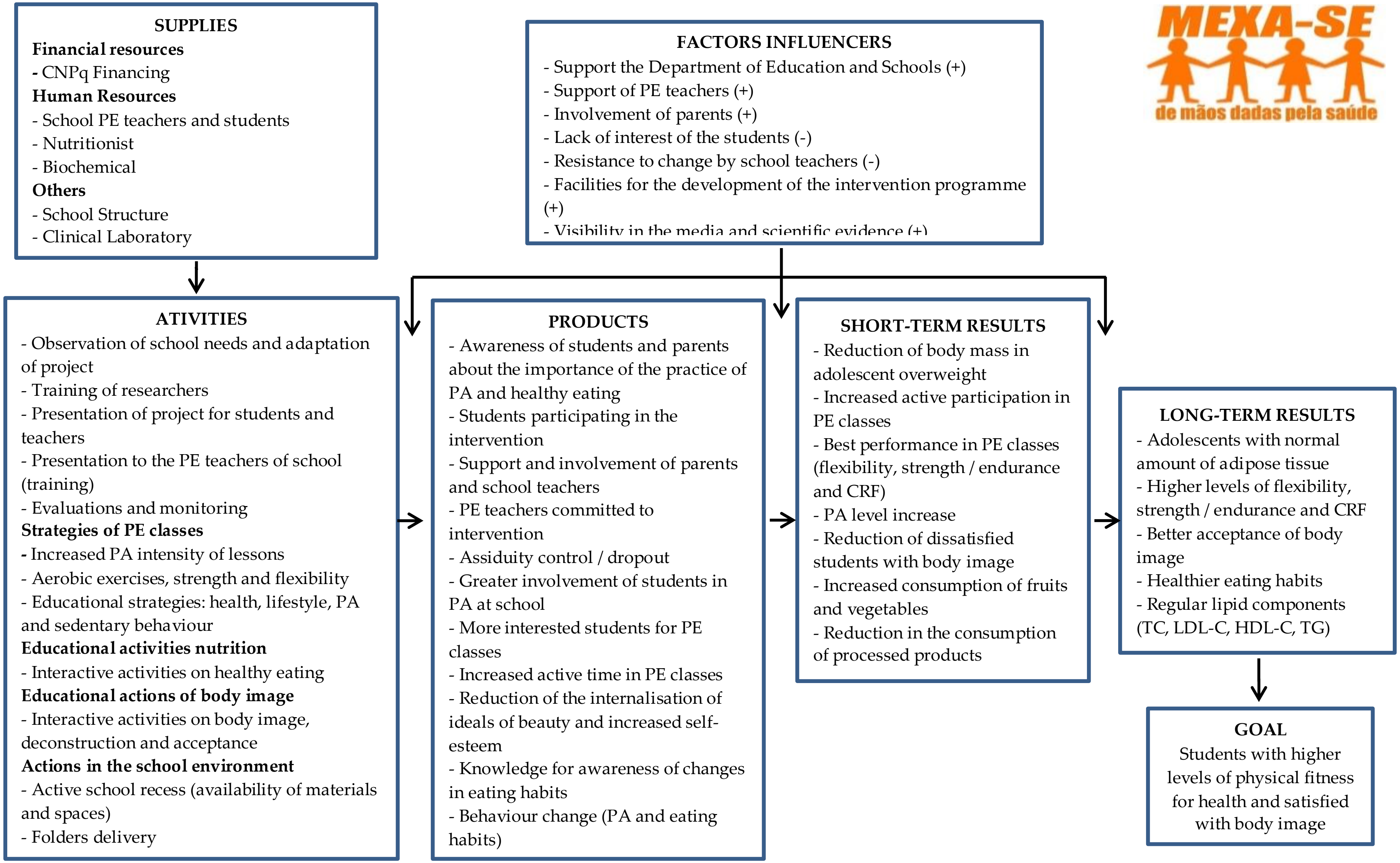

Figure S1. Logical Model of "*MEXA-SE*" (move yourself) intervention based of model USDHHS/CDC's Physical Activity Evaluation Handbook (2002).

Notes: CNPq: National Council for Scientific and Technological Development; PE: Physical Education; PA: physical activity; CRF: cardiorespiratory fitness; TC: total cholesterol; LDL-C: LDL-cholesterol; HDL-C: HDL-cholesterol, TG: triglycerides.

Figure S2. Pamphlets for parents: Physical Activity and Health.

Front

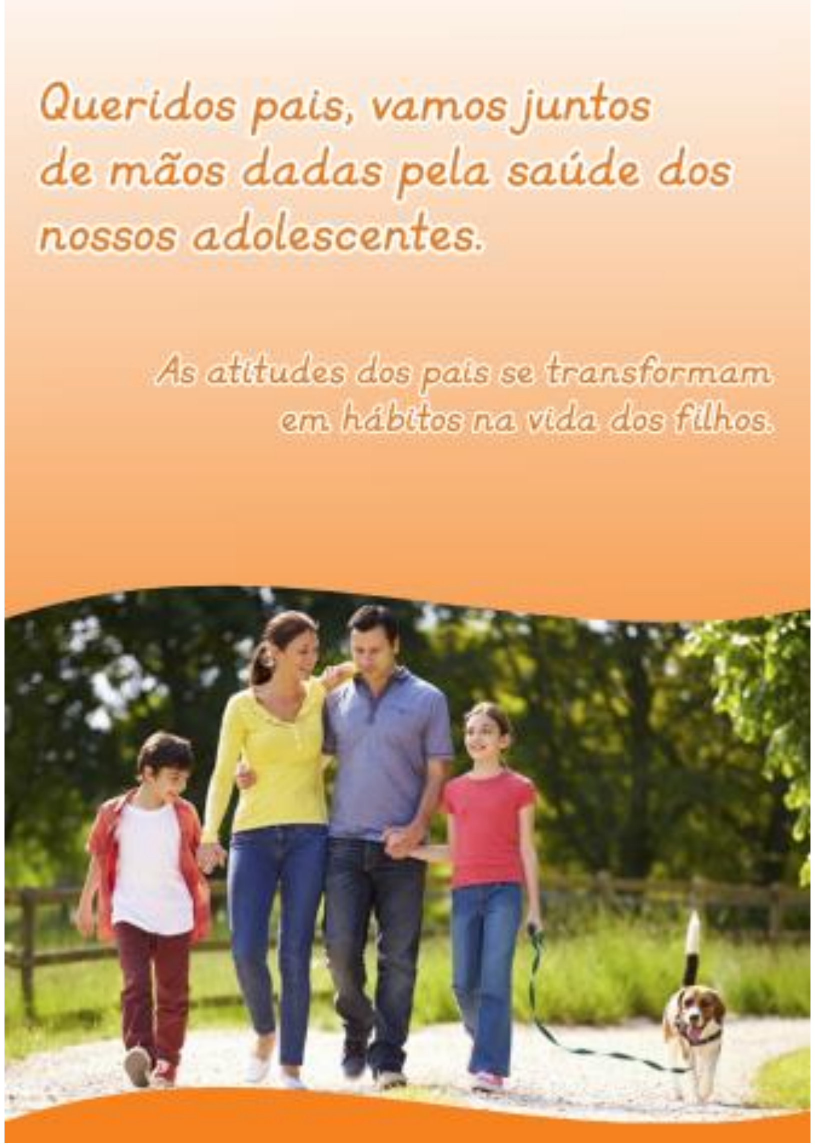

Back

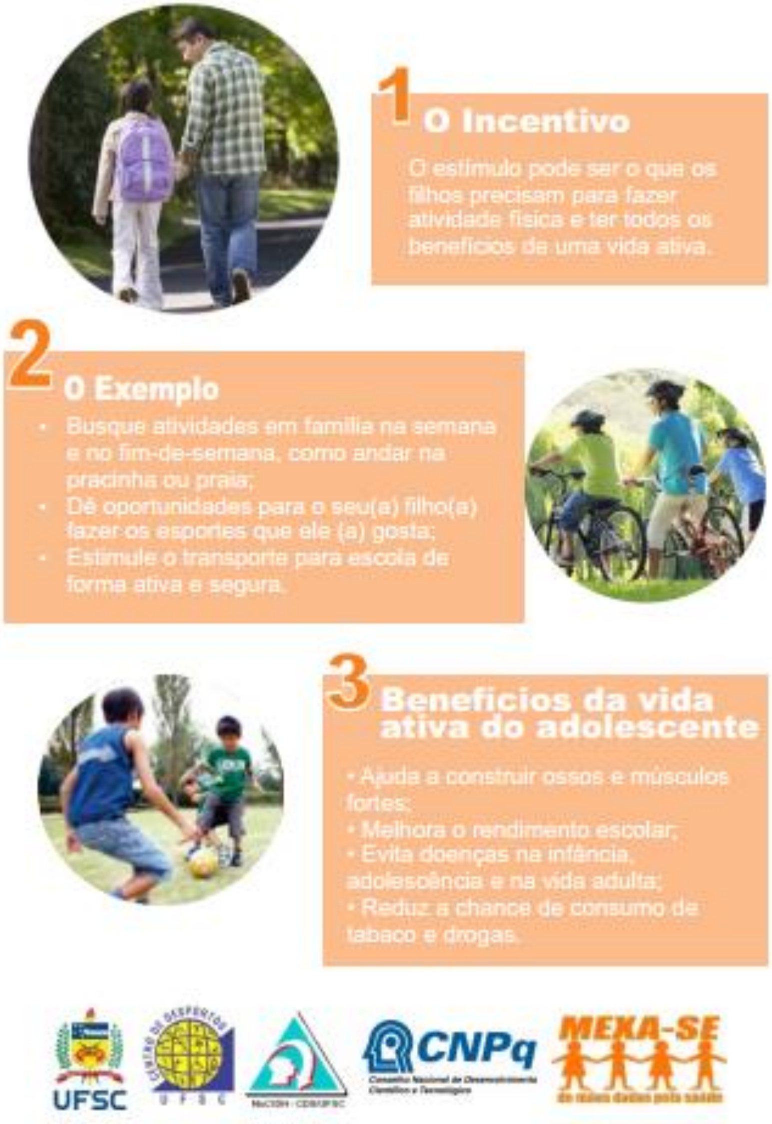

Pamphlets for parents: Sedentary Behaviour

Front

Quanto tempo seu(a) filho(a) passa, por dia, em atividades sedentárias, como assistir TV, usar o computador ou jogar videogame?

O tempo excessivo nestas atividades pode gerar alguns problemas à saúde do seu(a) filho(a), como:

- Obesidade e distúrbios metabólicos;
- Problemas no coração, como pressão alta e glicemia elevadas;
- Pode estimular comportamentos agressivos e o hábito de fumar, consumir bebidas alcoólicas e má alimentação;
- Menor concentração nos estudos e pior rendimento escolar;
- Menor interação com os amigos e com a família.

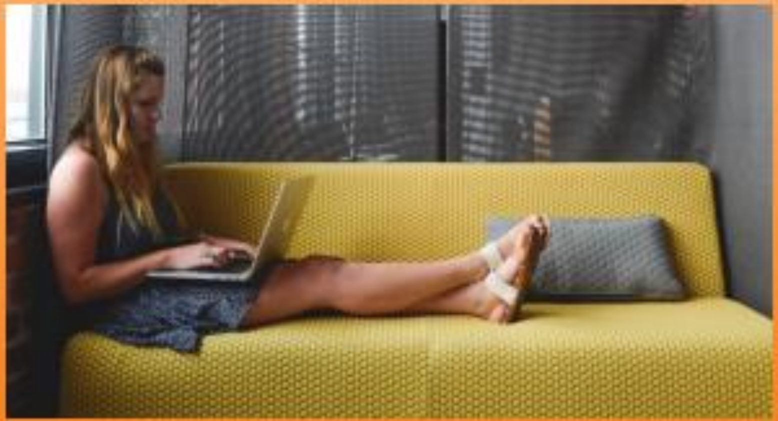

Back

QUANTO TEMPO?

No máximo duas horas por dia, somando o tempo de TV, computador, videogames e outros eletrônicos durante o lazer.

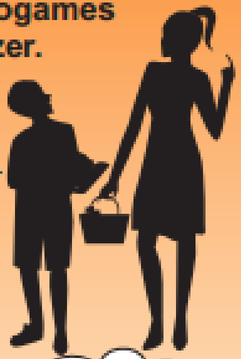

Outras dicas importantes:

- Evite que seu(a) filho(a) "navegue" no computador ou na TV. Se ele(a) não sabe o que vai assistir ou fazer, é porque não é tão interessante para ele(a);

- Sugira o tempo de TV executando outras tarefas físicas, como limpar a casa, lavar louças ou brincando ativamente;

- Escolha junto com seu(a) filho(a) o que ver e converse sobre o que ele(a) está acessando;

- Não deixe que seus filhos usem TV ou computador durante as refeições, aproveite para conviver em família;

- Por mais que o programa esteja interessante, faça pausas e MEXA-SE.

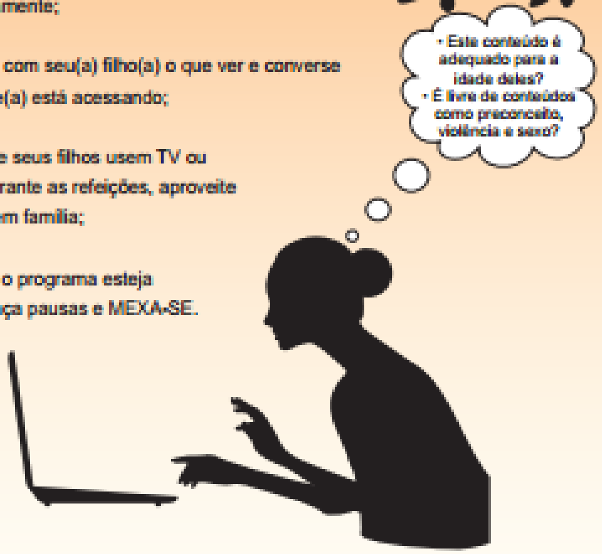

UFSC · CNPq · MEXA-SE

Figure S3. Pamphlets for students: Physical Activity and Health.

Front

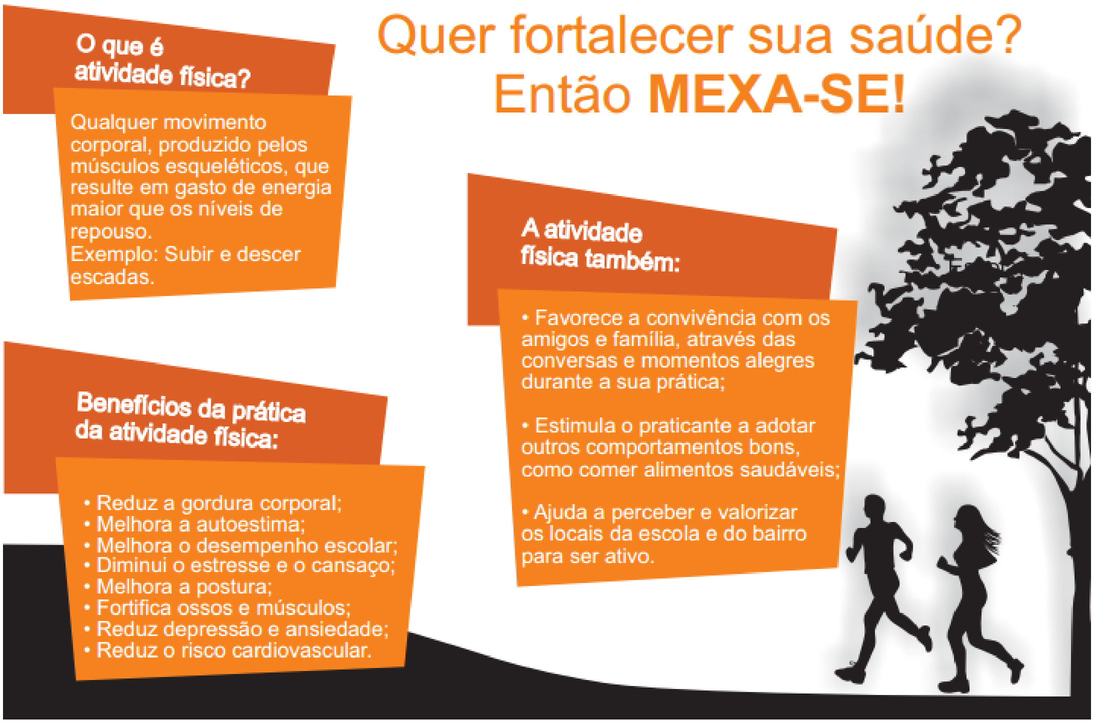

Back

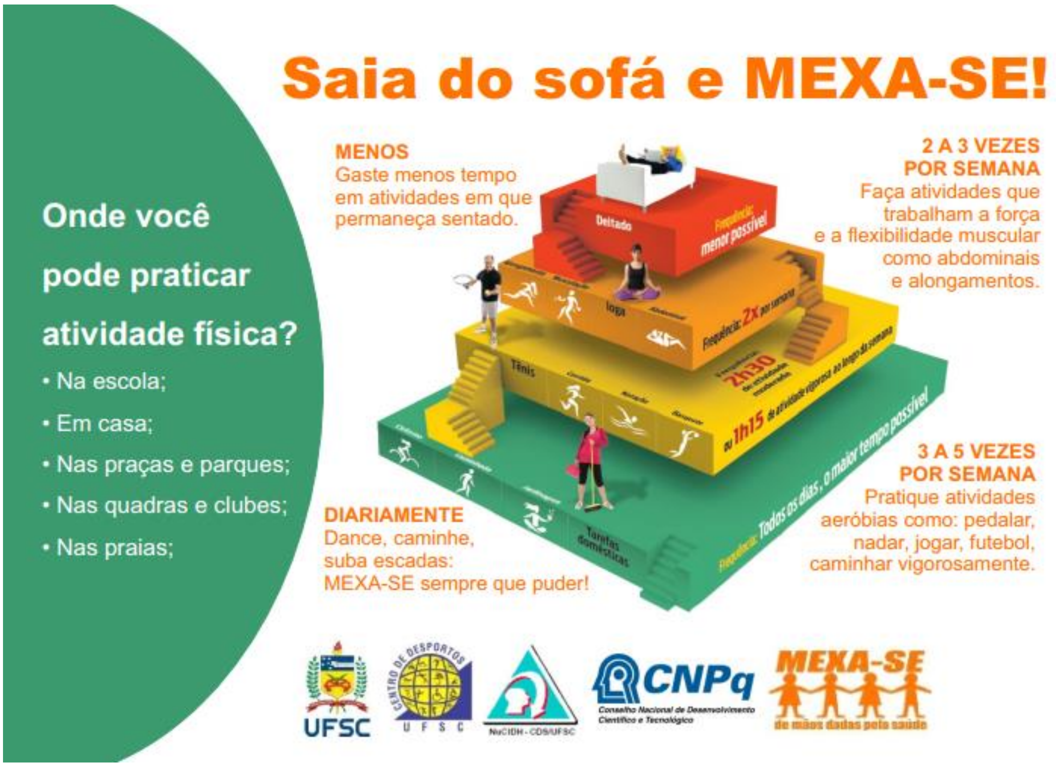

Pamphlets for students: Sedentary Behaviour

Front

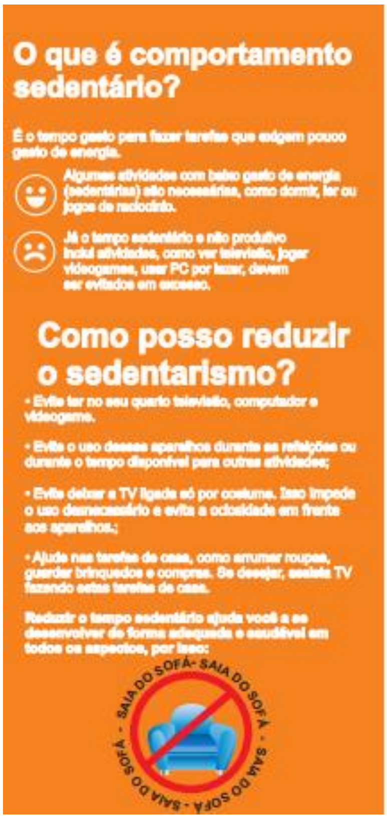

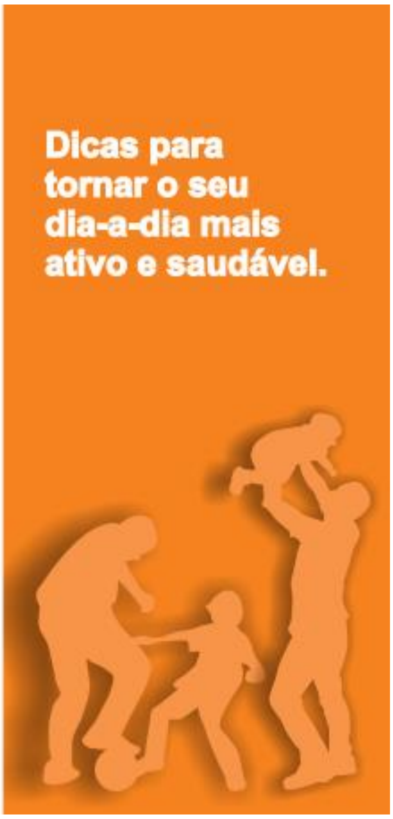

Back

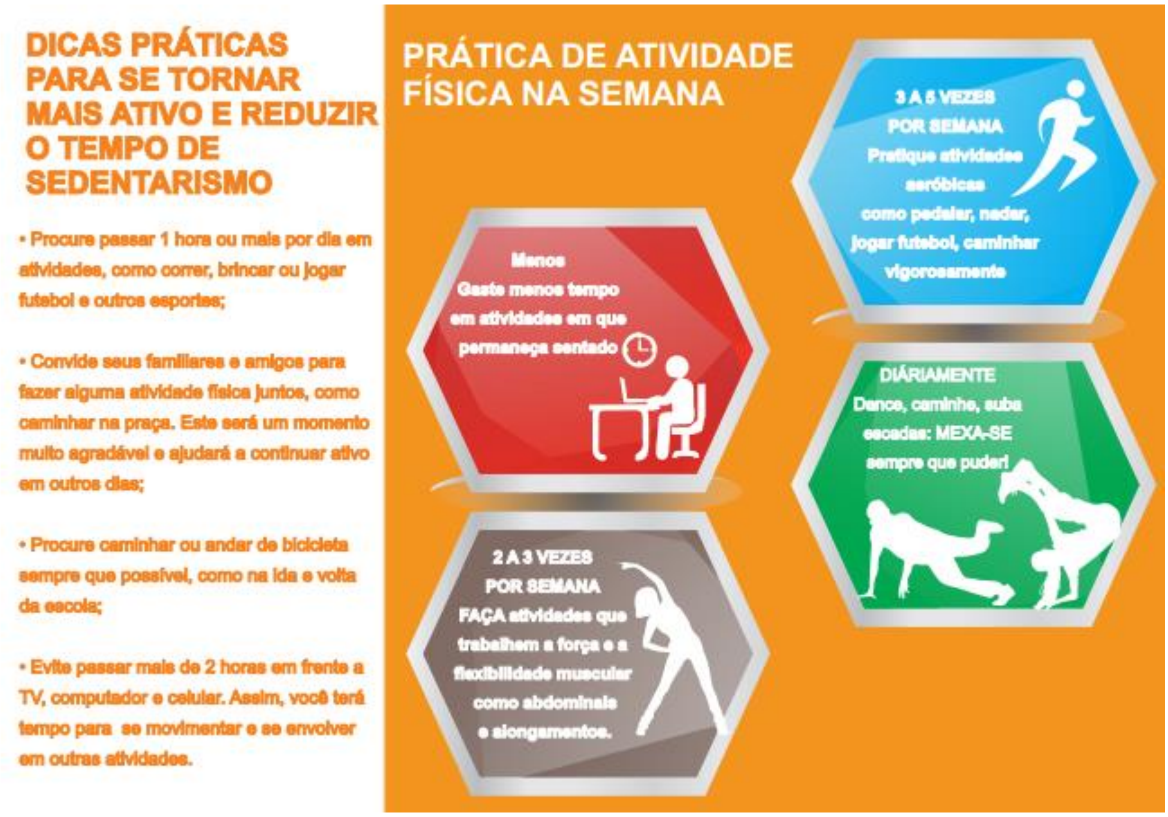

Supplement: Supplementary file 1 [file jfmk-04-00010-s001.pdf]
